# Navigating the Coronary Landscape: Towards Establishing Intracoronary Optical Coherence Tomography in Research and Clinical Practice

**DOI:** 10.31083/RCM52708

**Published:** 2026-08-11

**Authors:** Antonios Karanasos, Periklis Davlouros

**Affiliations:** ^1^Department of Cardiology, University of Patras, 26504 Patras, Greece

## 1. Introduction

Coronary angiography has been the cornerstone of invasive assessment of coronary artery disease, both for delineating coronary anatomy and for guidance during percutaneous coronary intervention (PCI). However, coronary angiography is limited to visualizing the coronary lumen and therefore cannot adequately assess arterial wall morphology and the effects of PCI. Intravascular imaging (IVI) shifted the paradigm from luminal assessment to morphological characterization of the coronary artery wall. Among IVI modalities, optical coherence tomography (OCT) stands out for its microscopic-level resolution (10–20 µm), thus providing important *in vivo* insights into the mechanisms of atherosclerosis, but also from a clinical standpoint, establishing diagnostic and therapeutic applications. The present editorial, accompanying this special issue dedicated to intravascular OCT, summarizes its growth as an established research and clinical tool, while highlighting future developments.

## 2. Evolution of OCT

The evolution of OCT is nicely summarized by Castaldi et al. [1]. Although intracoronary OCT was conceived in the early nineties and the first human studies were performed in the early 2000s [2], it was not until the development of frequency domain OCT (FD-OCT) that this modality established continuous growth. The transition to FD-OCT eliminated issues of earlier time-domain systems that required prolonged vessel occlusion, thus making OCT a practical tool for routine catheterization [3]. By leveraging its excellent diagnostic accuracy for plaque characterization and thrombus detection, and its potential to identify microstructures such as macrophages and cholesterol crystals, pilot studies have provided valuable insights into the pathobiology of atherothrombosis and vascular healing following PCI [4]. Simultaneously, standardization of imaging findings aided mainstream adoption by providing expert consensus regarding plaque characterization and stent assessment [5,6]. Based on this background, large-scale studies were performed, which proved the clinical benefit of OCT guidance [7], and provided the scientific basis for its eventual inclusion in international guidelines with a Class I, Level of Evidence A indication for PCI guidance in complex lesions [8,9,10,11].

## 3. Applications of OCT in Atherosclerosis Research and Acute Coronary Syndromes (ACS)

In patients with ACS, OCT has been used to investigate the association of plaque morphology and clinical presentation [12]. This has shifted the focus to the so-called vulnerable plaque as a potential target for ACS prevention [13]. In this context, two studies in this issue explore the relationship between systemic markers and local plaque characteristics, complementing existing knowledge regarding the interaction of inflammation with plaque morphology. Shi et al. [14] demonstrated that poor sleep quality (Pittsburgh Sleep Quality Index >9) and short sleep duration (≤5.5 h) are independent predictors of thin-cap fibroatheroma (TCFA), linking circadian disruption to plaque instability. In addition, Song et al. [15] found that inflammatory biomarkers—specifically high platelet-to-lymphocyte ratio (PLR) and low albumin—are independent predictors of coronary artery calcification, suggesting the presence of a metabolic-inflammatory axis in calcium deposition. These studies add to the current understanding of coronary atherosclerosis and help to further define the pathomechanisms of cardiovascular morbidity.

## 4. Clinical Applications of OCT

From a clinical aspect and relevant to this superior visualization of plaque microstructure, OCT is superior to angiography and intravascular ultrasound (IVUS) in identifying the underlying culprit mechanism of ACS, such as plaque rupture, plaque erosion, or calcified nodules [4]. This distinction is not merely academic. Synetos et al*.* [16] review how identifying plaque erosion may allow for tailored strategies, which include deferral of stent implantation in selected patients. Furthermore, in ambiguous angiographic morphology or challenging clinical scenarios such as myocardial infarction with non-obstructive coronary arteries (MINOCA) or spontaneous coronary artery dissection (SCAD), where angiography often fails to provide a diagnosis, OCT is an indispensable diagnostic tool.

Moreover, OCT has been established as an important tool for PCI guidance, especially in complex lesions [10,11]. Two studies in this special issue confirm the clinical utility of OCT in PCI guidance. The study by Li et al. [17] provides evidence for OCT guidance in ST-Elevation Myocardial Infarction (STEMI), a condition where randomized data is limited. This retrospective cohort of 1396 STEMI patients demonstrated a significantly increased risk of cardiovascular death with angiography-guided primary PCI compared to OCT guidance (adjusted hazard ratio 2.025; *p* = 0.004). Despite its limitations, including its retrospective design and the potential for selection bias, this study lends weak yet additional evidence in favor of imaging guidance even in the acute setting, pending further confirmation from randomized data. Mehmedbegovic et al*.* [18] investigated by OCT the long-term sequelae of angiography-guided left main PCI. In their study, long-term follow-up using OCT revealed high rates of malapposition (up to 13% in the polygon of confluence) and neoatherosclerosis (33%), emphasizing the critical need for imaging optimization during the index procedure to prevent late stent failure. This is a scenario where OCT has an established indication for identifying the pathology and mechanisms of stent failure and serves to guide future treatment [10,11].

## 5. The Implementation of Artificial Intelligence (AI) and Future Directions

The future of OCT lies in widespread adoption and automation. Considering that limited experience with OCT is often a barrier to a wider adoption, technological advances enabling fast, operator-independent assessment of plaque morphology and stent expansion can help overcome this problem. As highlighted by Sperti et al. [19], the integration of AI is transforming OCT analysis. Automated algorithms can now characterize plaque composition and identify vulnerable features (e.g., TCFA, macrophages) with increased speed and consistency that rival human expert interpretation, potentially integrating these insights into real-time clinical decision support systems (CDSS). Importantly, the integration of AI represents a step toward standardization and may reduce inter-observer variability associated with human interpretation of complex images [20].

## 6. Current Status of Intravascular OCT—a Critical Appraisal

Currently, intravascular OCT has established roles both in research and clinical practice. From a clinical perspective, established diagnostic indications include ambiguous angiographic morphology in ACS, where OCT is indispensable and part of the diagnostic algorithm in MINOCA [21]. Assessment of lesion severity by OCT in chronic coronary syndromes is not as well established as the current standard of invasive measured physiological assessment (fractional flow reserve [FFR] or non-hyperemic pressure ratios, such as iFR) [22]. Nevertheless, given the growing adoption of angiography-derived wire-free indices of ischemia, the use of OCT-derived physiological indices may increase the diagnostic yield of OCT in this context. In PCI guidance, the impact of OCT appears to be similar to IVUS; the decision to choose one modality over another may differ according to the clinical setting. For example, there is an advantage of IVUS in aorto-ostial lesions, chronic total occlusions, or renal insufficiency, and for OCT in ACS, stent failure, or bifurcation lesions. Nonetheless, the striking improvements in outcomes demonstrated by IVI need to be weighed against logistical issues, such as cost and increased procedural duration. From a health system perspective, IVI appears to be associated with acceptable costs for the anticipated benefits in life expectancy and quality-adjusted life years [23]. Yet, the benefit of IVI guidance is more prominent in complex lesions [24], suggesting a need to better refine the patient subsets that would result in greater benefit. One of these subsets is stent failure, where the role of IVI is pivotal both for the identification of the pathology and mechanisms, and for guiding individualized treatment. However, the interventionalist should maintain a low threshold for using IVI in the presence of unexpected difficulty.

Looking ahead, the convergence of technologies, such as hybrid IVUS-OCT or OCT-near-infrared spectroscopy catheters, polarization-sensitive OCT, and AI-derived FFR, promises a “one-stop” modality for anatomical, biological, and physiological assessment [1]. As we embrace these advancements, the interventionalist’s role shifts from assessing the lumen to understanding the complex vessel wall biology. This special issue underscores that OCT is far from a diagnostic luxury; it is indispensable for achieving optimized, patient-specific interventional outcomes.
